# Investigation of the Theragnostic Role of KIT Expression for the Treatment of Canine Mast Cell Tumors with Tyrosine Kinase Inhibitors

**DOI:** 10.3390/vetsci11100492

**Published:** 2024-10-10

**Authors:** Davide De Biase, Marcello De Leo, Giuseppe Piegari, Ilaria d’Aquino, Evaristo Di Napoli, Carmela Mercogliano, Alfonso Calabria, Agata Pula, Luigi Navas, Valeria Russo, Orlando Paciello

**Affiliations:** 1 Department of Pharmacy, University of Salerno, 84084 Fisciano, Italy; ddebiase@unisa.it; 2 Department of Veterinary Medicine and Animal Production, University of Naples Federico II, 80138 Napoli, Italyluigi.navas@unina.it (L.N.); valeria.russo@unina.it (V.R.);

**Keywords:** canine mast cell tumor, tyrosine kinase inhibitors, biomarkers

## Abstract

**Simple Summary:**

Canine mast cell tumors (MCTs) are a neoplasm frequently diagnosed in dogs that have a very variable aggressive behavior. The aim of our research was to assess the potential role of KIT in canine mast cell tumors in term of response to therapy by investigating the association between KIT expression patterns, the cumulative survival and the progression-free survival in dogs treated with tyrosine kinase inhibitors postoperatively. Dogs that underwent surgery alone were used as a control population. A log-rank test was used to check the differences between the curves, showing that tumors with KIT staining pattern 3 were significantly associated with decreased CS compared to tumors with KIT patterns 2 and 1. Our data suggest that the anomalous expression of KIT is negatively associated with the efficacy of tyrosine kinase inhibitors, thus providing a meaningful prognostic information about the treatment outcome.

**Abstract:**

Several reports have indicated that canine MCTs express a mutated form of a tyrosine kinase receptor, namely KIT, that is involved in abnormal mast cell growth and differentiation. Currently, the post-surgical prognosis for MCTs is related to three different KIT immunohistochemical expression patterns. However, to our knowledge, there are few studies specifically exploring the efficacy of treatment with tyrosine kinase inhibitors related to KIT staining pattern. The purpose of this study was to investigate the potential theragnostic role of KIT expression patterns by studying their correlation to the overall survival and progression-free survival in dogs treated with only tyrosine kinase inhibitors immediately after surgery. We selected 66 cases of canine cutaneous MCTs with complete clinical background. A statistical analysis was performed to assess the overall survival status. Our data suggest an important role of KIT in the etiopathogenesis of canine MCTs and indicate that the anomalous cytoplasmatic distribution of KIT is potentially related to a lower efficacy of tyrosine kinase inhibitors, thus providing a significant prognostic information about the treatment outcome.

## 1. Introduction

Canine mast cell tumor (MCT) is a neoplasm frequently diagnosed in dogs that has a very variable aggressive behavior [1,2]. The etiology of canine MCTs is still not completely elucidated, but the stem cell factor receptor KIT has an important and well-recognized role in the development of MCTs [1,3]. KIT is a tyrosine kinase receptor encoded by the proto-oncogene *c-kit* that has a pivotal role in the differentiation, proliferation, migration and survival of mast cells [3,4,5,6]. Three different patterns of immunohistochemical expression of KIT protein in neoplastic canine mast cells have been extensively described [3]; immunohistochemical expression and protein localization of KIT in neoplastic cells and, more importantly, histologic grade are currently the most informative markers for the prognostication of canine MCTs [3,7,8,9,10,11]. When there is no evidence of metastasis, surgery is the treatment of choice for solitary MCTs [12], but systemic targeted chemotherapy is also indicated when surgical excision with adequate margins is not feasible [1,12,13] To date, the most successful approach has been the use of a class of drugs termed “small molecule tyrosine kinase inhibitors” (TKIs) [14,15]. These molecules typically work by blocking the ATP-binding site of kinases, acting as competitive inhibitors that may be reversible or irreversible [16,17,18]. In the absence of ATP binding, the kinase is not able to phosphorylate itself or initiate downstream signaling [16]. To date, two orally bioavailable RTK inhibitors are approved by the *European Medicines Agency and the United States Food and Drug Administration* for treating canine MCTs [14,15,16]. Among them, Toceranib was approved for treating recurrent, non-resectable, grade II and III MCTs (according to the Patnaik morphological grading system) [14], while Masitinib has been approved for the same group of tumors when these harbor *c-kit* mutations [14]. Numerous studies have focused on the investigation of predictive factors for MCT outcomes and prognosis [17,18,19,20]. However, in veterinary medicine, there is limited data on clinically reliable markers that can predict, with some degree of certainty, the efficacy of a specific targeted therapy. In light of these considerations, our study aimed to address the potential theragnostic role of KIT in canine MCTs in terms of response to therapy by investigating the association between KIT expression patterns, cumulative survival and progression-free survival in dogs treated with the tyrosine kinase inhibitors Toceranib phosphate (Palladia^®^, Zoetis Italia S.r.l., Rome, Italy) and Masitinib mesylate (Masivet^®^, AB Science S.A, Paris, France) after surgery compared to dogs that underwent surgery alone.

## 2. Materials and Methods

### 2.1. Study Overview and Case Inclusion Criteria

We performed a retrospective cohort study on primary cutaneous MCTs submitted to the Veterinary Pathology Laboratory of the Department of Veterinary Medicine (University Federico II of Naples, Italy) in a time range of over 4 years (2019 to 2023). Medical records were collected for each case and included complete clinical staging, serum biochemistry profile and full blood count, physical examination, regional lymph node palpation with or without aspiration cytology, abdominal ultrasound and treatment. Lymph nodes were only considered positive for lymph node metastasis if they contained clusters or sheets of mast cells. The case selection criteria included (1) confirmed histologic diagnosis of canine cutaneous MCT according to the 2-tier histologic grading system proposed by Kiupel et al. [19]; (2) complete excision of the tumor with no invasion of the margins; (3) immunohistochemical analysis for KIT expression; (4) complete history and follow-up data obtained by the owner in the form of a questionnaire or telephone interview; (5) complete staging; (6) absence of severe concurrent disease; (7) no concurrent systemic anti-neoplastic therapy other than tyrosine kinase inhibitors and/or radiation therapy; and (8) absence of measurable disease immediately following surgery. Follow-up information was obtained from 6 to 48 months post-surgery and included anamnestic data; localization and size of the tumor; dates of additional tumor development or metastasis confirmed by clinical, cytological or histological examination; cause of death; and status at the last examination. Based on their medical reports and history, the animals were divided into two groups, as follows:

Group A—dogs treated with either Palladia (Toceranib phosphate), 3.25 mg/kg orally, every other day or Masivet^®^ (Masitinib mesylate), 12.5 mg/kg orally, every other day for six weeks. Dose reductions and dose interruptions for up to 2 weeks were permitted to manage adverse events.

Group B—dogs with canine MCTs treated with surgery alone (control population). Group B dogs were used to compare the outcomes between dogs treated with surgery alone and those treated with surgery and postoperative chemotherapy with a TKI.

### 2.2. Immunohistochemistry and Evaluation of KIT Staining Patterns

Immunohistochemistry for the evaluation of KIT expression patterns was performed using a well-established protocol described elsewhere [18]. Briefly, 4 μm thick sections of formalin-fixed and paraffin-embedded canine MCTs were mounted on silane-coated glass slides (Bio-Optica, Milan, Italy). A heat-induced epitope retrieval (HIER) was performed as an antigen retrieval pretreatment, submerging the slides in citrate buffer pH 6.0 (Bio-Optica, Milan, Italy) for 20 min at 98 °C. Next, endogenous peroxidase (EP) activity was reduced using 3% hydrogen peroxide (H_2_O_2_) in methanol. Then, the sections were treated with a protein block (MACH1, Biocare Medical LLC, Concord, CA, USA) for 30 min each. The slides were incubated overnight at 4 °C with the primary antibody diluted 1:200 in PBS (0.01 M PBS, pH 7,2). The primary antibody used for the study was Polyclonal Rabbit Anti-Human CD117/KIT (DAKO, Dako Italia S.p.A. Via Piero Gobetti, Milan, Italy). A DAB chromogen diluted in a DAB substrate buffer was used to visualize the antigen-antibody reaction, and the slides were counterstained with hematoxylin. Between all incubation steps, the slides were washed two times (5 min each) in PBS. The primary antibody was either omitted or substituted with a 1:20 dilution of rabbit serum (Code 011-000-120, Jackson Immuno Research, 872 West Baltimore Pike, West Grove, PA, USA) for the negative control sections. According to the method of Webster et al., we identified 3 patterns of KIT protein localization, as previously described for canine cutaneous MCTs [7,18]. The MCTs chosen for this study presented strong expression of KIT in at least 10% (estimated based on 100 neoplastic cells in a high-power field) of the neoplastic cells. Cells on the margins of the tissue sections were not considered due to possible artifactual staining.

### 2.3. Statistical Analysis

The statistical analysis was performed using the program SPSS Version 22.0 (IBM Corporation, 2014, Armonk, NY, USA). Cumulative survival (CS) and progression-free survival (PFS) were analyzed and measured in months from the date of the surgery. Treatment with surgery alone or surgery followed by chemotherapy with a TKI and KIT staining patterns were considered as the variables assessed for the analysis. Kaplan–Meier survival curves were plotted. A log rank was used to compare the survival curves, and a *p*-value of <0.05 was considered statistically significant.

## 3. Results

### 3.1. Dogs’ Demographics and Clinical Background

A total of 66 cases fit our selection criteria and were included in this study (Table 1). The median age of the patients at presentation was 8 years. Males and females were similarly represented in our cohort, with 34 female (17 spayed and 17 intact) and 32 male (10 neutered and 22 intact) dogs. The selected cases represented 19 breeds, including mixed-breed dogs (n. 29, 43%), Labrador retrievers (n. 9, 13%), Boxers (n. 3, 4%), Sharpei (n. 3, 4%), Maltese dogs (n. 3, 4%), Setters (n. 5, 7%) and 13 other breeds, which were represented by single animals (19%). Tumors were grossly identified as single, nodular masses ranging from 4 to 12 cm in diameter and localized mostly on the trunk (85%) and rarely on the limbs (10%) and face (5%). Of the 66 dogs, 33 underwent surgery and received chemotherapy with a TKI (22 with Masitinib mesylate and 11 with Toceranib phosphate) (Group A) and 33 underwent surgery alone (Group B). Complete staging information was available for all the patients. To compare similar populations of animals, all the selected MCTs were not recurrences of previous excisions. Moreover, clinical staging performed for all the MCTs by radiographs, ultrasound and regional lymph node cytology did not reveal the presences of metastasis to the lymph nodes or internal organs (HN0 stage) immediately before surgery.

### 3.2. KIT Staining Patterns and Dogs’ Survival Status

The results of the immunohistochemical analysis and the number of cases for each variable related to KIT staining pattern are summarized in Table 2.

**Figure 1:**
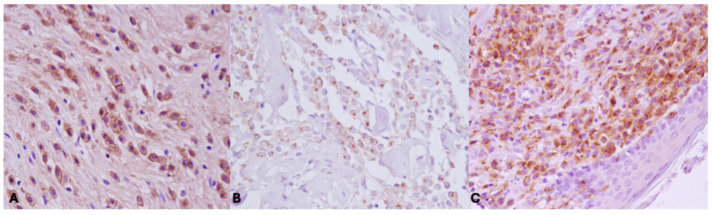
KIT immunohistochemical staining patterns. (**A**) Pattern 1, identified by membranous labeling in more than 90% of neoplastic cells. (**B**) Pattern 2, identified by focal, perinuclear or stippled cytoplasmic labeling with loss of perimembranous labeling in more than 10% of neoplastic cells. (**C**) Pattern 3, identified by diffuse cytoplasmic labeling in more than 10% of neoplastic cells.

A total of 27 MCTs (40%) presented a membranous KIT staining pattern (KIT pattern 1), while 21 MCTs (31%) showed focal, paranuclear cytoplasmic staining (KIT pattern 2) and 18 (27%) MCTs had diffuse cytoplasmic staining (KIT pattern 3) (Figure 1). Of the 33 dogs treated with post-surgery chemotherapy with a TKI (Group A), 9 (27%) showed KIT staining pattern 1, 13 (39%) showed KIT staining pattern 2 and 11 (33%) showed KIT staining pattern 3. The median survival times were, respectively, 22.88 (SE 5.6), 27.15 (SE 5.7) and 7.63 (SE 0.95) months.

**Figure 2:**
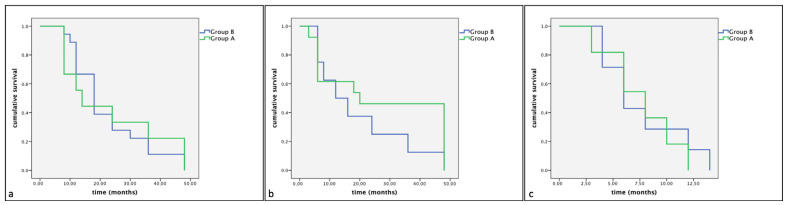
Kaplan–Meier survival graph comparing the cumulative survival times in the three KIT expression subclasses: (**a**) pattern 1, (**b**) pattern 2 and (**c**) pattern 3.

**Figure 3:**
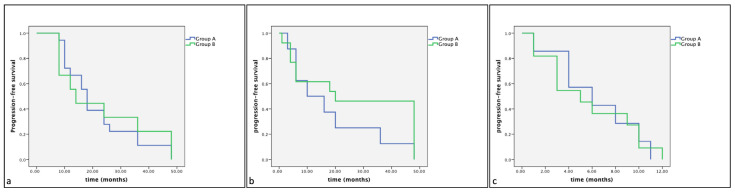
Kaplan–Meier survival graph comparing the progression-free survival times in the three KIT expression subclasses: (**a**) pattern 1, (**b**) pattern 2 and (**c**) pattern 3.

Of the 33 dogs treated with surgery alone (Group B), 18 (54%) showed KIT staining pattern 1, 8 (24%) showed KIT staining pattern 2 and 7 (21%) showed KIT staining pattern 3. The mean survival times were, respectively, 22.33 (SE 2.93), 19.50 (SE 5.46), 7.17 (SE 1.47) months. No statistically significant differences were observed between groups (Group A: dogs treated with Palladia^®^ or Masivet^®^; Group B: dogs treated with surgery alone) in the same KIT expression subclasses (Kit pattern 1, Kit pattern 2 and Kit Pattern 3) in terms of cumulative survival and progression-free survival (Figure 2 and Figure 3).

**Figure 4:**
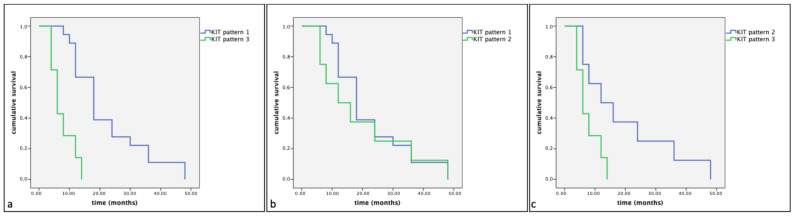
Kaplan–Meier survival graph comparing the three KIT staining patterns in term of cumulative survival times in dogs with MCTs treated post-surgery with TKI therapy. (**a**) KIT pattern 1 vs. KIT pattern 3; (**b**) KIT pattern 1 vs. KIT pattern 2; (**c**) KIT pattern 2 vs. KIT pattern 3.

**Figure 5:**
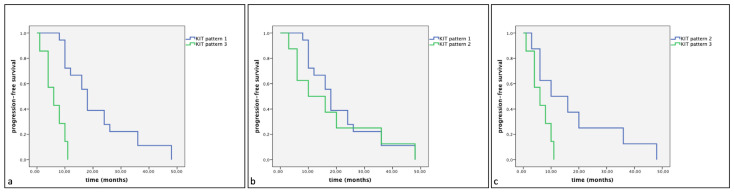
Kaplan–Meier survival graph comparing the three KIT staining patterns in term of progression-free survival times in dogs with MCTs treated post-surgery with TKI therapy. (**a**) KIT pattern 1 vs. KIT pattern 3; (**b**) KIT pattern 1 vs. KIT pattern 2; (**c**) KIT pattern 2 vs. KIT pattern 3.

**Figure 6:**
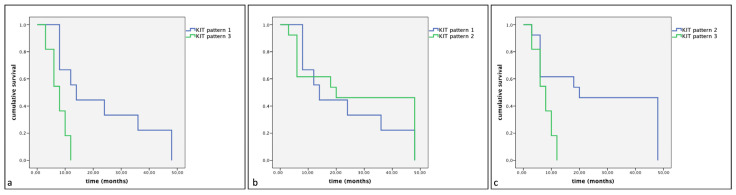
Kaplan–Meier survival graph comparing the three KIT staining patterns in term of cumulative survival times in dogs with MCTs treated with surgery alone. (**a**) KIT pattern 1 vs. KIT pattern 3; (**b**) KIT pattern 1 vs. KIT pattern 2; (**c**) KIT pattern 2 vs. KIT pattern 3.

**Figure 7:**
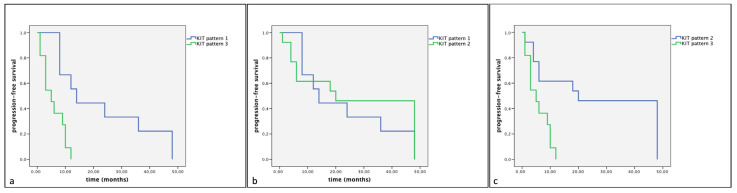
Kaplan–Meier survival graph comparing the three KIT staining patterns in term of progression-free survival times in dogs with MCTs treated with surgery alone. (**a**) KIT pattern 1 vs. KIT pattern 3; (**b**) KIT pattern 1 vs. KIT pattern 2; (**c**) KIT pattern 2 vs. KIT pattern 3.

However, a statistically significant difference in cumulative survival times and progression-free survival (*p* < 0.05) was observed between the different KIT expression patterns. Indeed, the Kaplan–Meier and log-rank tests displayed a significantly shorter cumulative survival and progression-free survival in Group A in dogs showing KIT expression pattern 3 compared to those showing KIT expression pattern 1 (*p* < 0.01) and KIT expression pattern 2 (*p* < 0.01) (Figure 4 and Figure 5). Similarly, Group B dogs showed KIT staining pattern 3 compared to dogs showing a KIT pattern 1 (*p* < 0.01). Furthermore, differences were observed between KIT pattern 3 and KIT pattern 2 in the same assessed group (*p* < 0.05) (Figure 6 and Figure 7).

## 4. Discussion

In the scientific literature, there are several interesting and breakthrough studies focused on the role of the KIT staining pattern as a prognostic factor in dogs treated with chemotherapy, such as vinblastine and prednisone [20] or tyrosine kinase inhibitors [21]. In this retrospective study, we investigated the theragnostic potential of KIT expression patterns for tyrosine kinase inhibitor treatments in canine MCTs, analyzing as a final outcome both the cumulative survival (CS) and progression-free survival (PFS). Toceranib phosphate (Palladia^®^) and Masitinib mesylate (Masivet^®^) are tyrosine kinase inhibitors that are currently approved for companion animals and indicated for the target therapy of canine mast cell tumors. Masitinib mesylate and Toceranib phosphate have a very similar mechanism of action, both exerting their effect by blocking cross-phosphorylation of intracellular tyrosine residues by binding to the ATP-binding site of the catalytic domain of KIT [22]. The consequence of this binding is the termination of the downstream intracellular signaling required for the survival and growth of malignant mast cells, followed by cell cycle arrest and apoptotic cell death [22,23]. Despite their similarities, Toceranib phosphate and Masitinib mesylate have slightly different affinities and sensitivities; indeed, Toceranib simultaneously targets multiple tyrosine kinase receptors such as KIT, Vascular Endothelial Growth Factor Receptor (VEGFR), Platelet-Derived Growth Factor Receptor (PDGFR) and Colony-Stimulating Factor 1 (CSF-1) receptor. On the other hand, Masitinib mesylate has a more specific inhibitory activity for KIT, even though it may also target other tyrosine kinase receptors, including PDGFRs and Fibroblast Growth Factor Receptor 3 (FGFR3) [24]. Our data showed that TKIs such as Masitinib mesylate (Masivet^®^) and Toceranib phosphate (Palladia^®^) have significantly and progressively lower efficacy in MCTs showing KIT staining pattern 3, pattern 2 and pattern 1. Not surprisingly, similar results were observed in dogs that underwent surgical resection of the primary lesion not followed with the administration of TKI-based chemotherapy. The results of this study further validate the KIT staining pattern as a prognostic factor and suggest its theragnostic potential because the aberrant cytoplasmic distribution of KIT gives significant information about the possible treatment outcomes. We speculate that the cytoplasmic aberrant expression of KIT (pattern 3) may be related to a lower drug efficiency and subsequent lower efficacy of TKI-based chemotherapy. One of the most described mechanisms by which a tumor does not respond to a specific targeted therapy is the reactivation of the target kinase for either the amplification of the target gene or secondary mutations in the kinase [25,26]. It has been described that this additional mutation occurs most often in the target kinase domain, drastically altering the drug-binding affinity by (1) modifying the amino acids surrounding the binding site, thereby diminishing the ability of the drug to reach its target, or (2) perturbing the contact point between the drug and the target [27,28,29,30]. The altered KIT expression may be related to *c-kit* mutations, even though mutations are not present in all MCTs with aberrant KIT expression [5]. In human neoplasms, *c-kit* mutations have been indicated to provoke constitutive (independent from its ligand) KIT phosphorylation and activation by impairing the regulatory functions of the juxtamembrane domain and by directly targeting the kinase domain [25,26,27]. Such mutations are expected to be the reason for the increased cell proliferation in MCTs exhibiting cytoplasmic KIT expression [25,26,27]. Gil da Costa et al. [31] speculated that mutations causing constitutive KIT phosphorylation may also collide with the intracellular traffic of KIT and cause the molecule to be stored in cellular organelles, such as the endoplasmic reticulum or Golgi apparatus. A profounder comprehension of the role of KIT in tumorigenesis may conceivably be obtained by clarifying the exact cellular localization of KIT accumulations within the cytoplasm, especially in lesions presenting a focal, perinuclear pattern (pattern 2). Studies focused on gastrointestinal stromal tumors (GISTs) in humans described cases showing acquired resistance to a targeted therapy with imatinib that have been reported and molecularly analyzed [32]. Although numerous mechanisms have been suggested to be accountable for this phenomenon, including KIT gene amplification, the occurrence of additional point mutations seems to be the most recurrent event suggesting that imatinib secondary resistance in GIST could be dependent on a phenomenon not yet completely explored in terms of temporal development of the tumor [33]. The point mutations secondary to chemotherapy that have been described until now always comprise the kinase domain of the receptor and have been described in exons 13, 14 and 17 of the *c-kit* gene and in exon 18 of the *PDGFRA* gene [34,35]. Their presence in the ATP pocket of the kinase indicates that imatinib is not able to efficiently inhibit the receptor, almost certainly owing to a dissimilar tridimensional protein structure of the ATP pocket induced by these additional mutations [35]. An interesting topic for further investigations could be the research for a specific mutation of *c-kit* inducing conformational changes in the KIT activation loop and the development of a molecular model that may demonstrate the drug–protein interaction and subsequently the drug efficacy.

### Limitations

Because of its retrospective nature, this work has important limitations that need to be addressed. First, we could not standardize the risk-based groups (KIT staining pattern and histological grade) with an equal and consistent number of cases and, most of all, the two different TKIs used to properly compare the efficacy of the treatment (with surgery alone or with post-surgical chemotherapy with either Masitinib mesylate or Toceranib phosphate) for each KIT expression pattern. Second, the limited number of cases for each group may have negatively influenced the statistical results about the efficacy of the two different treatments (TKI or surgery alone), causing a false negative (statistical type II error). Moreover, we considered the treatment with two different tyrosine kinase inhibitors as a single group, even though they have different sensitivities to KIT. Finally, the lack of information about the *c-kit* mutation status before and after the chemotherapy with a TKI was another limitation of this study. In our opinion, prospective studies that include more cases and more standardized groups are needed (1) to reduce the likelihood of a type II error; (2) to further evaluate the hypothesis that aberrant cytoplasmic KIT localization (pattern 3) could have a major pathologic relevance representing a feature of activated kinase mutants that could alter the efficacy of tyrosine kinase inhibitors and predispose to drug resistance; (3) to focus on the expression of KIT in tumor recurrences to figure out if the pattern of expression changes or not; and (4) to improve a precision medicine approach by evaluating the efficacy of a single TKI in relation to the outcome and the intrinsic characteristics of the patient, mutational status and the tumor. Nowadays, several veterinary pathologists and oncologists suggest that the decision to begin treatment with a TKI should be made concerning the results of the PCR for activating mutations in exons 8 and 11 of *c-kit* and the pattern of KIT expression, as evaluated by immunohistochemistry (IHC). We fully embrace the conclusions of Sledge et al. [36] suggesting a holistic approach to the prognostication and treatment of canine MCTs that combines clinical assessment, histopathologic evaluation and molecular diagnostics.

## 5. Conclusions

This study aimed to investigate the potential prognostic role of KIT expression patterns in terms of response to therapy with tyrosine kinase inhibitors. The results of this study further support the use of KIT protein localization for the prognostication of canine MCTs. Furthermore, our results suggest that intracellular localization of the KIT protein is strongly related to a diminished efficacy of targeted therapy with tyrosine kinase inhibitors. The log-rank test was used to check the differences between curves showing that tumors with KIT staining pattern 3 were significantly associated with decreased CS and PFS compared to tumors with KIT patterns 2 and 1. Our data suggest that the aberrant expression of KIT is negatively associated to the efficacy of tyrosine kinase inhibitors, thus providing meaningful prognostic information about the treatment outcome.

## Figures and Tables

**Table 1:** Dog’s demographics.

Cases Characteristics	Value
Total participants	66
Median age at presentation (years)	8 (range 1–14)
Gender	
Female spayed	17
Female intact	17
Male neutered	10
Male intact	22
Breed	
Mixed breeds	29
Labrador retriever	10
Sharpei	3
Boxer	3
Setter	5
Maltese	3
Other breeds	13
Histologic grade (by Kiupel)	
Low	34
High	22
Number of metastatic diseases	
Group A (TKI)	3
Group B (Surgery alone)	4
Number of recurrences	
Group A (TKI)	1
Group B (Surgery alone)	3

**Table 2:** Results of the immunohistochemical analysis.

Variable	KIT Staining Pattern 1	KIT Staining Pattern 2	KIT Staining Pattern 3	Value
Immunohistochemistry	27	21	18	66
Grade				
High grade	6	7	9	22
Low grade	21	14	9	44
Treatment				
Group A (Toceranib phosphate)	2	5	4	11
Group A (Masitinib mesylate)	7	8	7	22
Group B (Surgery alone)	18	8	7	33

## Data Availability

All data used in the current study are available from the corresponding author on reasonable request.
